# Synergistic potential of CDK4/6 inhibitors and ATRA in non‐APL AML

**DOI:** 10.1111/bjh.70057

**Published:** 2025-08-04

**Authors:** Rafał Skopek, Setenay Gupse Özcan, Paulina Chmiel, Stephanie Morgner, Jacqueline Schütt, Faezeh Ghazvini Zadegan, Clara Stanko, Małgorzata Palusińska, Karolina Maślińska‐Gromadka, Yordan Sbirkov, Sven Stengel, Martin Fischer, Annamaria Brioli, Arthur Zelent, Łukasz Szymański, Tino Schenk

**Affiliations:** ^1^ Department of Molecular Biology Institute of Genetics and Animal Biotechnology, Polish Academy of Sciences Magdalenka Poland; ^2^ Department of Hematology/Oncology Clinic of Internal Medicine II, Jena University Hospital Jena Germany; ^3^ Institute of Molecular Cell Biology, Center for Molecular Biomedicine Jena (CMB), Jena University Hospital Jena Germany; ^4^ Computational Biology Group, Leibniz Institute on Aging—Fritz Lipmann Institute (FLI) Jena Germany; ^5^ Department of Soft Tissue/Bone Sarcoma and Melanoma Maria Sklodowska‐Curie National Research Institute of Oncology Warsaw Poland; ^6^ Department of Internal Medicine C University Medicine Greifswald Greifswald Germany; ^7^ Hematology, Hemostasis, Oncology and Stem Cell Transplantation, Hannover Medical School (MHH) Hanover Germany; ^8^ Medical University of Plovdiv Plovdiv Bulgaria; ^9^ Research Institute at Medical University of Plovdiv Plovdiv Bulgaria; ^10^ Department of Neuropediatrics Jena University Hospital Jena Germany

**Keywords:** acute myeloid leukaemia, AML, ATRA, CDK4/6, CDK4i, non‐APL, palbociclib, ryuvidine, synergy

## Abstract

Acute myeloid leukaemia (AML) is a heterogeneous disease characterized by diverse genetic abnormalities. The standard of care remains to be chemotherapy and stem cell transplantation. In acute promyelocytic leukaemia (APL), differentiation therapy with all‐trans retinoic acid (ATRA) has significantly improved outcomes. Despite this, the success of ATRA has yet to be transferred to non‐APL AML. Exploring combinations to enhance the efficacy of ATRA in non‐APL AML remains a key focus. To investigate the therapeutic effect of ATRA in combination with cyclin‐dependent kinase 4/6 (CDK4/6) inhibitors in non‐APL AML. Non‐APL AML cell lines and primary patient samples were treated with ATRA and CDK4/6 inhibitors. Key outcomes included differentiation, proliferation, cell viability and colony‐forming capacity. Combination synergy was evaluated, and gene expression analysis identified pathways associated with therapeutic effects. The combination demonstrated dose‐dependent effects, enhancing differentiation and reducing proliferation, cell viability and colony‐forming capacity. A synergistic effect was observed across AML cell lines. Gene expression profiling revealed the co‐regulation of differentiation‐associated genes, unveiling the mechanisms driving therapeutic synergy. Combination of CDK4/6 inhibitors with ATRA shows potential for differentiation‐based AML treatment. This approach offers a promising avenue for improved outcomes in non‐APL AML.

## INTRODUCTION

Acute myeloid leukaemia (AML) is a disease with high degree of clinical and genetic heterogeneity.^1^ AML subtypes that exhibit recurrent genetic abnormalities account for approximately 20%–30% of AML cases.^2^, ^3^, ^4^ The multifactorial malignant transformation of AML has a direct impact on long‐term treatment response and survival.^5^ Despite advances in targeted therapy, 30%–40% of patients experience recurrence, which leads to poor prognosis.^6^, ^7^, ^8^, ^9^ All‐trans retinoic acid (ATRA) has emerged as a revolutionary agent in differentiation therapy for acute promyelocytic leukaemia (APL).^10^ The combination with arsenic trioxide (ATO) resulted in highly favourable treatment outcomes in the majority of patients with *PML::RARA* translocation.^11^ As a ligand for retinoic acid receptors (RARs), ATRA induces the formation of heterodimeric complexes with retinoic X receptors (RXRs), which initiate granulocytic differentiation.^12^, ^13^, ^14^ However, despite the lack of mutations in RARs, the success of ATRA does not extend to non‐APL AML, suggesting the existence of additional mechanisms blocking differentiation.^15^, ^16^, ^17^, ^18^, ^19^ Our previous work showed that epigenetic changes related to histone demethylation and acetylation contribute to ATRA resistance in other AML subtypes, indicating that combinational targeting of diverse signalling pathways could sensitize AML cells to retinoic acid.^17^, ^20^, ^21^

The mutations in the *CCND1* and *CCND2* (cyclin D1/D2) genes are frequent in AML.^22^, ^23^ D‐type cyclins form complexes with cyclin‐dependent kinase 4 (CDK4) and cyclin‐dependent kinase 6 (CDK6) to induce the transition from the G1 to the S phase of the cell cycle.^24^ Recently, CDK4/6 inhibitors (palbociclib and ribociclib) have become standard components of management in advanced breast cancer, as favourable outcomes were reported in clinical trials.^25^, ^26^ Palbociclib and ribociclib are ATP‐competitive inhibitors of CDK4/6, which exert their effects through the inhibition of retinoblastoma protein (RB) phosphorylation, consequently causing cell cycle arrest in the G1 phase.^25^ Ryuvidine, a known antifungal compound, has also been shown to inhibit CDKs, with a certain degree of specificity towards CDK4.^27^, ^28^

Considering previous reports on the impact of CKD4/6 inhibitors and their clinical efficacy,^29^, ^30^, ^31^ we hypothesized that targeting the cell cycle with the CDK4/6 inhibitor palbociclib augments ATRA‐induced differentiation in AML cells. We evaluated the effects of palbociclib, ryuvidine and a specific CDK4 inhibitor (CDK4i) in combination with ATRA on AML cell lines and primary samples. We aimed to assess the impact of these combination therapies on cell proliferation, differentiation and viability and to elucidate the underlying molecular mechanisms.

## MATERIALS AND METHODS

### Cell culture

The cell lines were purchased from American type culture collection (ATCC) (HL‐60, KG‐1) and German collection of microorganisms and cell cultures (DSMZ) (OCI‐AML2, OCI‐AML3, THP‐1, MV4‐11, MOLM‐13). Cells were maintained as recommended by vendors. Patient samples were obtained after informed consent in accordance with the Declaration of Helsinki. The study was approved by the Institutional Review Board of University Hospital Jena. Blasts were isolated by density gradient separation (Lymphoprep, STEMCELL Technologies) and treated as indicated. ATRA and palbociclib (PD‐0332991 isethionate) were purchased from Sigma‐Aldrich, ryuvidine and CDK4 inhibitor (CDK4i) were purchased from Cayman Chemical.

### Flow cytometric analysis of differentiation and cell cycle

Cells were treated for 3 days alone or in combination with the drugs. After treatment, 1 × 10^6^ cells were harvested and stained with fluorescein isothiocyanate (FITC)‐labelled anti‐human CD11b antibody (clone ICRF44) and APC/Cy7‐labelled anti‐human CD14 antibody (clone M5E2) (Biolegend) in PBS with 1% FCS and analysed with BD LSRFortessa™.

Propidium iodide was used for the cell cycle analysis. After 3‐day treatment, the cells were washed with PBS and then fixed with 70% ice‐cold ethanol. Subsequently, the cells were stained with PI in PBS (12.5 μg/mL) and treated with RNase A (200 μg/mL) for 30 min at 37°C, then analysed with BD FACS Canto™. All flow cytometry experiments were analysed with FCS Express 5 Research edition or FlowJo (Tree Star) software.

### Cytospin preparation

1.5 × 10^5^ HL‐60 cells were seeded in six‐well plates and treated alone or in combination with ATRA and palbociclib for 5 days. Subsequently, the cells were harvested and centrifuged at 550 rpm for 5 min with Shandon Cytospin 4 centrifuge. The slides were air‐dried overnight at room temperature and fixed by immersion in methanol for 5 min. Pappenheim staining (May–Grünwald & Giemsa, Morphisto) was carried out according to the manufacturer's protocol. The samples were analysed by Nikon Eclipse Ti‐E inverted microscope in conjunction with NIS‐Elements 5.01 software.

### Oxidative burst assay

To assess functional differentiation, the oxidative burst assay was performed. Cells were treated for 5 days, followed by incubation with 10 μg/mL Phorbol‐12‐myristate‐13‐acetate and 1 mg/mL nitroblue tetrazolium (NBT, Sigma‐Aldrich) at 37°C for 1 h. Cells were then fixed with ice‐cold methanol and lysed with 0.9 M potassium hydroxide (KOH) and 54% dimethyl sulfoxide (DMSO). The absorbance was measured with a Tecan Infinite® F200 plate reader at 595 nm.

### Immunoblotting

2 × 10^5^ cells per well seeded in a 12‐well plate, treated with 1 μM ATRA and 500 nM palbociclib alone or in combination for 24 h. Cells were lysed with radioimmunoprecipitation assay (RIPA) buffer supplemented with protease inhibitor cocktail and phosphatase inhibitors (Roche). Protein concentrations were determined using the Pierce™ BCA Protein Assay Kit (Thermo Scientific). The lysates were separated using sodium dodecyl sulfate polyacrylamide gel electrophoresis (SDS‐PAGE) and western immunoblotting, as previously described.^18^ Antibodies used for the assay are shown in Supporting Information.

### RNA sequencing

HL‐60 cells were single or double‐treated (3 h; 500 nM palbociclib, 100 nM ATRA) and total RNA was isolated. TruSeq RNA Library preparation was performed using oligo(dt) beads binding all mRNAs by their polyadenylic (polyA) tail. Barcoded triplicates of each treatment condition including untreated were sequenced using Illumina NGS (HiSeq 2500).

For the RNA sequencing of patient samples, peripheral blood samples were obtained from 28 AML patients prior to treatment. Following peripheral blood mononuclear cells (PBMC) isolation, total RNA isolation, library preparation and NGS were performed as described above.

Detailed information regarding RNA‐seq analyses is provided in the Supporting Information.

### Statistical analysis

Data are represented as the mean ± standard deviation and were generated from three independent experiments unless otherwise specified. Statistical analysis was performed using two‐way analysis of variance (ANOVA) with Bonferroni post‐test using GraphPad Prism 10 software. Statistical significance was defined as *p* < 0.05 (*p < 0.05; **p < 0.01; ***p < 0.001).

Further information regarding analyses is provided in the Supporting Information.

## RESULTS

### The CDK4/6 inhibitor palbociclib, in combination with ATRA, increases the differentiation response in AML cells

**FIGURE 1:**
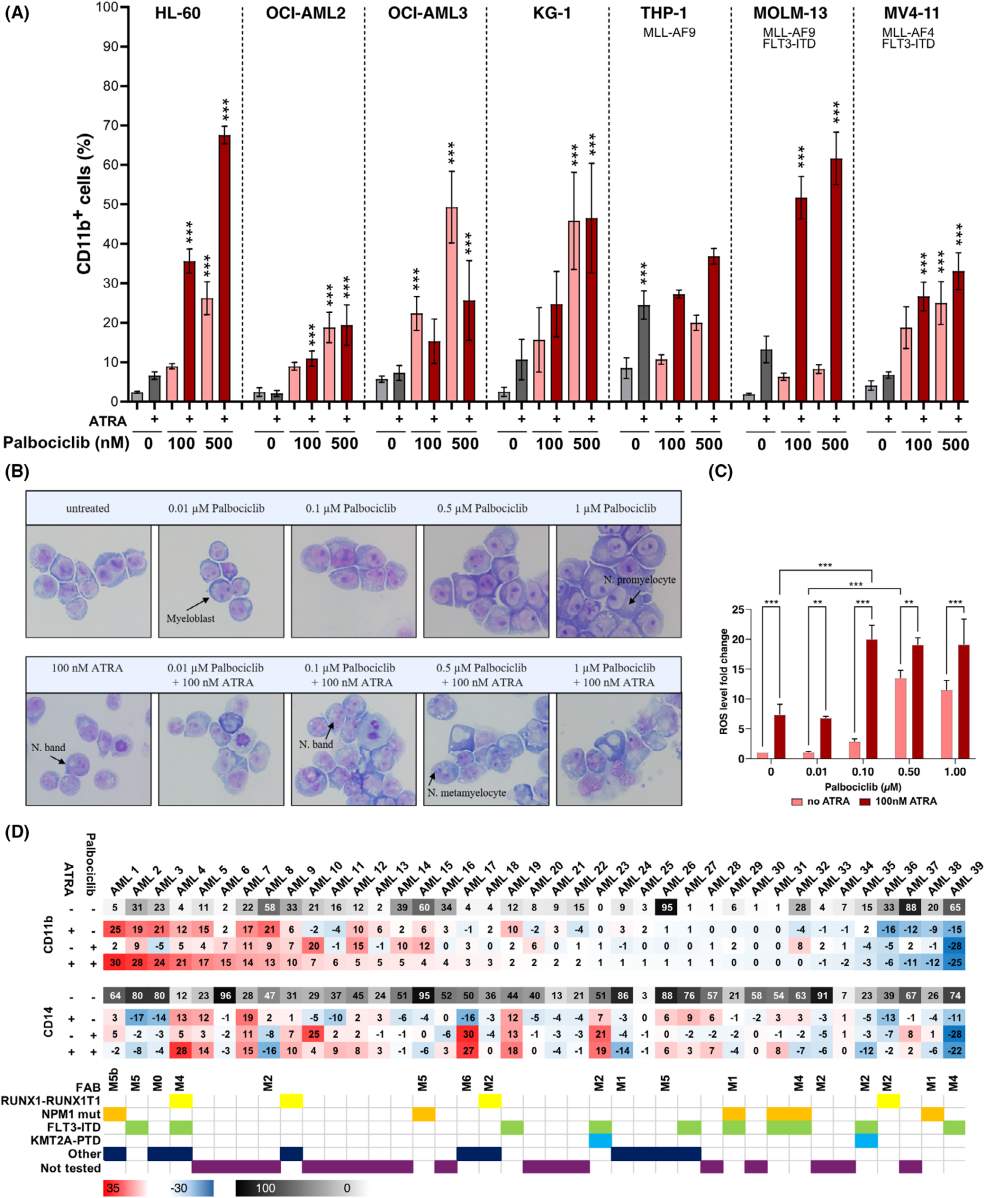
(A) Flow cytometric analysis of CD11b expression in AML cell lines treated with 500 nM palbociclib, ATRA (10 nM for THP‐1, 1000 nM for KG‐1 and 100 nM for the remaining cell lines) or in combination for 3 days. Statistical analyses were performed by comparing combined treatments to single ATRA treatments, whereas single palbociclib and single ATRA treatments were compared to untreated samples (mean ± SD, *n* = 3, two‐way ANOVA, Bonferroni correction). (B) Microscopic analysis of representative May–Grünwald–Giemsa‐stained cytospins from HL‐60 cells treated with palbociclib and ATRA for 5 days (magnification objective ×40 and ×10). (C) Nitroblue Tetrazolium assay showing the effects of palbociclib and ATRA on HL‐60 ROS production. Normalized to untreated controls. The combined treatments were compared with non‐ATRA treatments for statistical significance evaluation (**p < 0.01; ***p < 0.001). Other comparisons were indicated by brackets (mean ± SD, *n* = 3, two‐way ANOVA, Bonferroni correction). (D) Changes in differentiation markers CD11b and CD14 in patient AML samples after treatment with 1 μM ATRA, 500 nM palbociclib and in combination. The upper row in each heat map (grey scale) shows the percentage the indicated marker in untreated cells. The lower rows (red‐blue) show the percentages of cells that change expression following treatment. Selected recurrent genetic aberrations and French–American–British (FAB) classification of patient samples are presented in the lower panel.

We treated seven AML cell lines for 3 days to evaluate the joint effect of palbociclib and ATRA on differentiation response. Analysis of CD11b revealed a dose‐dependent increase after palbociclib treatment in all cell lines. Treatment with 500 nM palbociclib alone resulted in CD11b expression between 10% and 50%. Combination with ATRA enhanced palbociclib's effect across all cell lines, except OCI‐AML2 and OCI‐AML3, while prominent CD11b elevation was observed in HL‐60 and MOLM‐13 (Figure 1A).

Since palbociclib inhibits CDK4 and 6, to identify the predominantly responsible CDK, we treated the cell lines with a CDK4 inhibitor (CDK4i) and ryuvidine, which has weaker CDK4 inhibitory activity. Treatment with 500 nM ryuvidine induced a modest increase in CD11b expression (Figure S1A). CDK4i and ATRA combination yielded a stronger response in FLT3‐ITD cell lines MOLM‐13 and MV4‐11 compared to others except HL‐60 (Figure S2A). In HL‐60 cells, 1000 nM CDK4i with ATRA elevated CD11b, though weaker than palbociclib. CDK6 appears to contribute more to the effect in most cell lines tested, though more studies would be needed for a definitive conclusion.

Given the pronounced induction of CD11b by palbociclib in combination with ATRA, we further investigated this effect in HL‐60 cells (Figure 1B). Our histomorphological analysis revealed that individual treatment with palbociclib and ATRA did not induce noticeable morphological changes in HL‐60 cells, which retained their characteristic blast appearance. Although occasional cells suggested an increase in granularity, distinct differentiated cell types, such as neutrophils, were rarely observed.

To clarify this discrepancy between CD11b expression and morphology, we performed a nitroblue tetrazolium (NBT) assay to validate the functional differentiation of HL‐60 cells (Figure 1C). Single treatment with 100 nM ATRA increased reactive oxygen species (ROS) production up to sevenfold compared to untreated cells. The effect of ATRA was significantly augmented upon combination with 100 nM palbociclib. Higher concentrations of palbociclib with ATRA did not result in a further increase in ROS generation. Nevertheless, the combination induced significantly elevated ROS levels compared to both single‐drug treatments, which validates that the drug combination induces functional differentiation of HL‐60.

To corroborate our findings, we also treated 39 samples from AML patients with 1 μM ATRA and 500 nM palbociclib and evaluated CD11b and CD14 expression with flow cytometry (Figure 1D, patient characteristics are shown in Table S3). In primary cells, ATRA treatment alone yielded up to 24.7% increase in CD11b compared to the untreated baseline, while palbociclib showed a moderate increase, up to 10.5%. Depending on the AML subtype, differentiation manifested as an increase in CD14. Overall, the differentiation response was mainly patient‐dependent, which indicates the importance of evaluating patients on an individual basis to determine those that would potentially benefit from palbociclib.

### Dual treatment with ATRA and palbociclib results in cell cycle arrest and decline in clonogenic capacity

**FIGURE 2:**
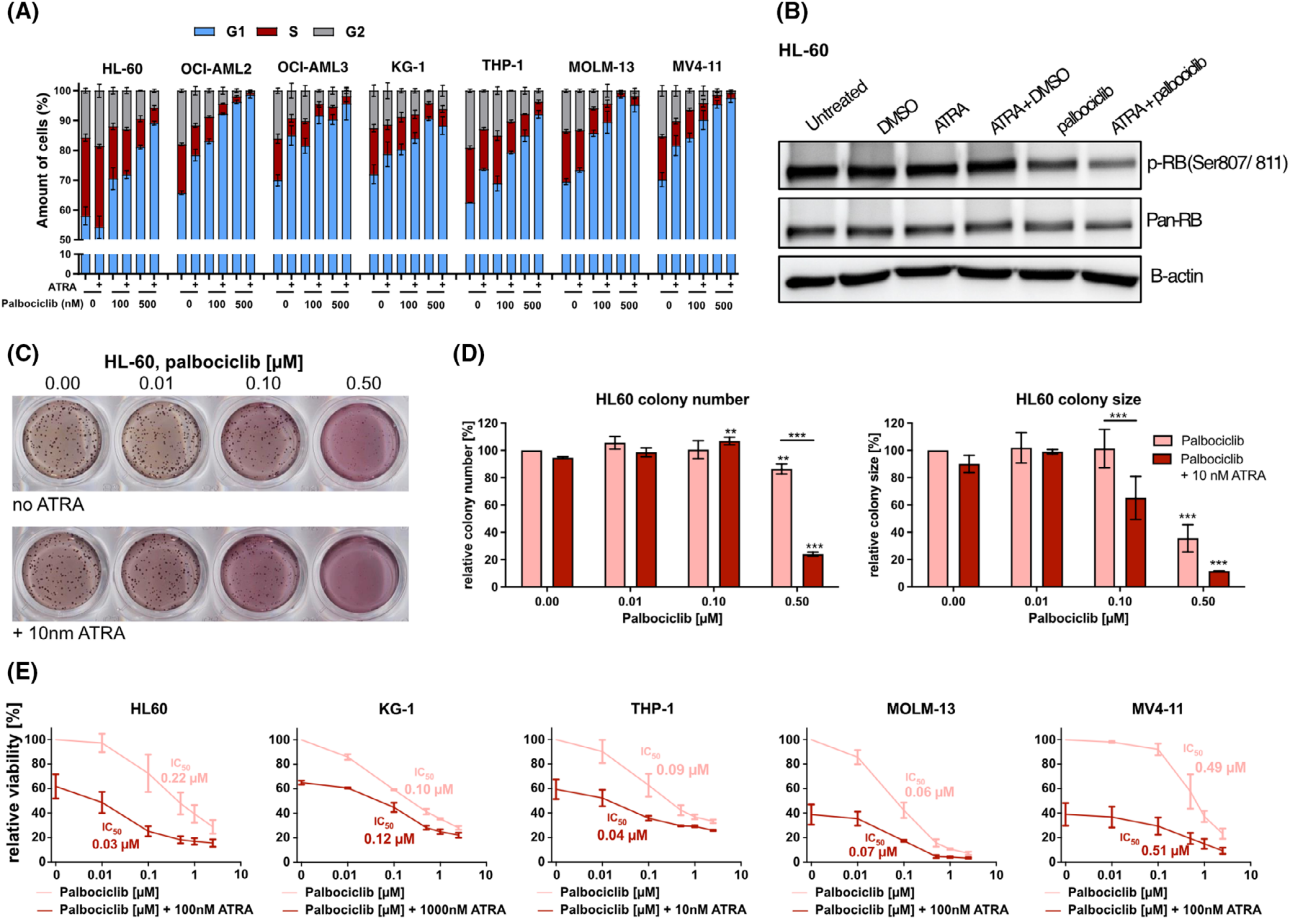
(A) Flow cytometric analysis of the cell cycle in AML cell lines treated with palbociclib (100 or 500 nM) and/or ATRA (10 nM for THP‐1, 1000 nM for KG‐1 and 100 nM for the remaining cell lines) for 3 days. (B) Analysis of Rb protein expression and phosphorylation in HL‐60 cells treated with 1‐μM ATRA, 500‐nM palbociclib or 0.05% DMSO (vehicle control) for 24 h. The panels show an expression of pan‐Rb, Serine807/811‐phosphorylated Rb and B‐actin (loading control). (C) Representative image of colony formation of HL‐60 cells treated with palbociclib and ATRA. HL‐60 cells were seeded in Iscove's Modified Dulbecco’s Medium (IMDM) medium–methylcellulose mixture and treated with palbociclib (0.01/0.10/0.50 μM) and 10 nM ATRA. Results after 9 days of cultivation are shown. (D) Left: HL‐60 colony number normalized to untreated cells. Right: HL‐60 colony size normalized to untreated cells. (E) Cell viability analysis of AML cell lines treated with palbociclib and ATRA. AML cell lines were treated with palbociclib and ATRA for 3 days, and the 3‐(4,5‐dimethylthiazol‐2‐yl)‐2,5‐diphenyltetrazoliumbromid (MTT) assay was performed. Absorbance was measured at 570 nm. IC50 values of single palbociclib treatment and combination treatment are indicated (mean ± SD, *n* = 3; each experiment performed as triplicates, normalized to untreated). **p < 0.01; ***p < 0.001. AML, acute myeloid leukaemia; ATRA, all‐trans retinoic acid.

Sustaining proliferation and enabling replicative immortality are well‐established hallmarks of cancer.^32^ As a CDK4/6 inhibitor, palbociclib triggers cell cycle arrest in the G1 phase. Accordingly, flow cytometric analysis showed that palbociclib induced dose‐dependent cell cycle arrest across all seven tested cell lines following a 3‐day treatment (Figure 2A). Notably, ATRA also induced an initial G1 arrest, except for HL‐60. The combination of palbociclib and ATRA further increased the accumulation of cells in the G1 phase. While ryuvidine did not induce an effect on the cell cycle in any of the AML cell lines at the tested concentrations (Figure S1B), treatment with CDK4i led to dose‐dependent G1 arrest, but to a lower extent than palbociclib (Figure S2B). FLT3‐ITD cell lines MV4‐11 and MOLM‐13 appeared to respond more strongly to CDK4i.

CDK4/6 inhibition by palbociclib causes cell cycle arrest through preventing phosphorylation of tumour suppressor RB.^33^ Our western blot analysis showed that treatment with 500 nM palbociclib for 24 h decreased Ser807/811 phosphorylation of RB in HL‐60 cells (Figure 2B). While 1 μM ATRA alone did not affect phospho‐RB levels, dual treatment with ATRA and palbociclib further decreased phospho‐RB, potentially explaining the strong combination effect.

To investigate palbociclib and ATRA's impact on proliferation and clonogenic survival, we performed an in vitro colony formation unit (CFU) assay with HL‐60 cells. We observed a dose‐dependent reduction in colony number and size upon palbociclib treatment (Figure 2C). Although colonies decreased slightly, a significant reduction in size was observed at 500 nM palbociclib. The combination of ATRA and 100 nM palbociclib resulted in a significant reduction in colony size. The clonogenic capacity was most reduced by the 500 nM palbociclib and ATRA combination, showing an 80% decrease in number and a 90% decrease in size compared to untreated HL‐60 colonies.

To further analyse the synergistic potential of ATRA and CDK inhibitors, we employed the MTT assay with HL‐60, KG‐1, MOLM‐13, MV4‐11 and THP‐1 cells and calculated IC50 (Figure 2D) and coefficient of drug interaction (CDI) values (Table S1) after 3 days of treatment. Palbociclib reduced the viability of all tested cell lines in a dose‐dependent manner. For single palbociclib treatment, the IC_50_ values were between 0.06 and 0.5 μM. Notably, combination with ATRA strongly enhanced the cytotoxic effect. CDI calculations showed a synergistic effect of the two drugs in HL‐60, MOLM‐13 and MV4‐11 cells and an additive effect for THP‐1 and KG‐1 (Table S1). Ryuvidine only had an effect on cell viability at higher doses (>0.50 μM) (Figure S4). The synergistic effect in combination with ATRA was detected solely in MOLM‐13 cells (Table S2). Overall, our data illustrate that the combination of ATRA and palbociclib induces cell cycle arrest and a decline in cell viability. Moreover, we observed a reduction in the clonogenic capacity and viability of HL‐60 cells, subsequently increasing the differentiation response.

### ATRA and palbociclib induce distinct and synergistic alterations in gene expression, enhancing differentiation and repressing proliferation‐related pathways

**FIGURE 3:**
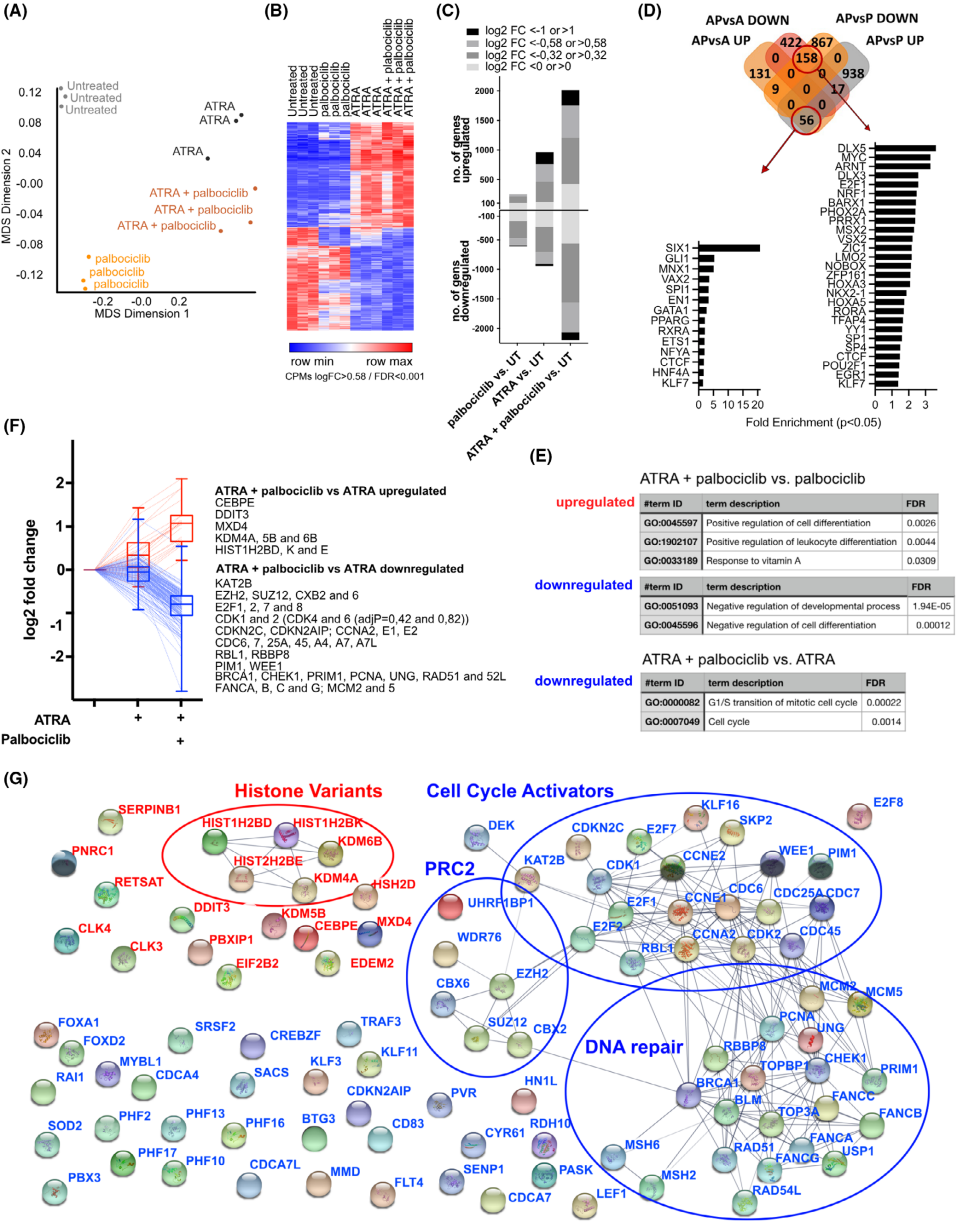
(A) Multidimensional scaling (MDS) plot showing similarities between RNA‐seq samples; HL‐60 cells, single or double‐treated (3 h; 500 nM palbociclib, 100 nM ATRA). (B) Heat map of unsupervised hierarchical clustering of genes differentially expressed in response to treatment. A red‐blue colour scale was used to reflect standardized gene expression, with red indicating higher expression and blue indicating lower expression. (C) Numbers of regulated genes following treatment relative to the untreated control. (D) Transcription factor enrichment analysis of genes exclusively regulated by the combination treatment of ATRA/palbociclib (AP) versus ATRA (A) and palbociclib (P) single treatments. (E) Gene ontology (GO) term enrichment analysis of differentially expressed genes. Only significantly (*p* < 0.05) enriched terms are shown. (F) Selected genes specifically upregulated (red) and downregulated (blue) by addition of palbociclib to ATRA. (G) Gene expression regulation by palbociclib + ATRA versus ATRA. Known and predicted protein interactions of upregulated (red) and downregulated (blue) genes were analysed by STRING (Search Tool for the Retrieval of Interacting Genes/Proteins, string‐db.org). Circles indicate protein clusters of distinct regulatory functions. ATRA, all‐trans retinoic acid.

To evaluate ATRA and palbociclib's combined effects on gene expression, we treated HL‐60 cells for 3 h with 500 nM palbociclib, 100 nM ATRA, or in combination, and performed RNA sequencing. Results revealed substantial synergy between treatments (Figure 3A–C). ATRA alone upregulated and downregulated almost 1000 genes each, with upregulated genes showing higher log2 fold‐change (log2 FC) values compared to the moderate suppression of downregulated genes. Palbociclib primarily downregulated genes and affected a smaller subset. The combination treatment induced or repressed over 2000 genes each, exceeding expected additive effects.

To investigate these effects, we identified genes uniquely upregulated or downregulated by combination treatment versus single treatments (Figure 3D). Transcription factor analysis showed the combination treatment activated differentiation‐associated nuclear receptor genes (e.g. RXRα, PPARγ) while suppressing proliferation‐related transcription factors (e.g. MYC, E2F1). Gene ontology term enrichment analysis revealed significant enrichment of retinoid‐mediated differentiation genes upregulated by ATRA, and enrichment of cell cycle‐associated genes in palbociclib‐downregulated gene sets (Figure 3E). Analysis of gene expression changes induced by adding palbociclib to ATRA (Figure 3F,G) highlighted clusters of upregulated histone variants and modifiers (e.g. KDM6B) and downregulated proteins involved in cell cycle progression, DNA repair and components of polycomb repressive complex 2 (PRC2). Examples include upregulation of genes such as CEBPE and histone‐encoding genes (HIST1H2BD, HIST1H2K) and downregulation of proliferation‐associated genes, including E2F1, EZH2 and CDK1. These findings underscore the enhanced regulatory effect of ATRA and palbociclib combination treatment, fostering differentiation while repressing proliferation and repair pathways critical to leukemogenesis.

To build upon our results, we analysed pretreatment primary samples from 28 AML patients to identify genes associated with therapy response. We correlated baseline gene expression to differentiation response to ATRA and palbociclib, measured by CD11b levels after ex vivo treatment. Among genes analysed, only 34 were expressed in at least two samples and analysed further. Our analysis revealed a high degree of correlation between the expression of individual genes. Consequently, the decision was made to proceed with the analysis of groups of correlated genes rather than individual genes, as illustrated in the heat map (Figure S4). A direct relationship was identified between the expression of *LTF*, *CRISP3*, *OLFM4*, *CEACAM5*, *PRUNE2*, *MLS*, *PADI2*, *GLRXP3*, *CRISP2*, *TRMT5*, *STAB2*, *MMP27*, *AMP32D*, *ACTA1*, *PPIAP26*, *LMNTD1* and *SYN2* (group number 1) and increased differentiation (*p* < 0.005).

## DISCUSSION

ATRA is central to APL therapy, yet its use in non‐APL AML remains limited due to resistance mechanisms that are not fully understood.^34^, ^35^, ^36^

Our study demonstrates that CDK4/6 inhibition represents a viable strategy to overcome this resistance and enhance differentiation in AML. We observed that combining ATRA with palbociclib significantly induced CD11b expression in multiple AML cell lines. Palbociclib alone increased CD11b by 10%–50%, while the addition of ATRA enhanced this effect up to 70% in HL‐60 cells. Although prior studies suggested that FLT3‐ITD or mixed lineage leukaemia (MLL) rearrangements may predict palbociclib responsiveness, our data did not confirm this in either cell line (Figure 1A) or primary AML samples (Figure 1D).^37^, ^38^

Cell cycle progression requires signals where CDK4/6 and Cyclin D mediate RB phosphorylation of RB and its siblings p107 and p130, leading to activation of cell cycle genes.^39^, ^40^, ^41^ Aberrations in this network contribute to leukaemic transformation through uncontrolled proliferation, making CDK4/6 a targetable mechanism.^37^, ^38^, ^42^, ^43^, ^44^, ^45^ Our analyses revealed palbociclib‐induced cell cycle arrest in AML cell lines, and combination with ATRA reduced clonogenic capacity and cell viability by up to 50% (Figure 2E). CDK4 inhibitor ryuvidine with ATRA also decreased cell viability, though requiring higher concentrations (Figure S4). In HL‐60 cells, palbociclib decreased RB phosphorylation, and while ATRA alone had no effect, combination treatment further reduced RB phosphorylation, which corroborates the observed synergy.

A key sign of ATRA‐induced differentiation in AML cell lines is neutrophil‐like morphological changes, including increased granularity and nuclear segmentation.^46^ In our study, despite strong CD11b induction with palbociclib and ATRA, morphological changes were minimal, showing mainly increased granularity (Figure 1B). Higher ATRA doses might enhance neutrophil‐like changes. However, functional differentiation was confirmed by a 19‐fold increase in ROS levels after ATRA and palbociclib co‐treatment, demonstrating their synergistic action.

Our findings align with recent work by Hu et al., who also reported synergy between ATRA and palbociclib via transcriptional changes involving RARA, E2F1 and STAT1.^47^ Moreover, in a recent case report, a female patient with therapy‐related refractory AML achieved complete remission after receiving palbociclib with venetoclax and azacitidine.^48^

RNA‐seq analysis highlighted extensive transcriptional reprogramming upon dual treatment. Genes regulating retinoid signalling and histone modification (e.g. KDM6B, CEBPE) were upregulated, while proliferation regulators (e.g. EZH2, E2F1, CDK1) were downregulated. Upregulated histone variants create an accessible nucleosomal landscape that synergizes with KDM6B's removal of H3K27me3 to activate differentiation genes.^49^, ^50^ Downregulation of EZH2 reduces H3K27 trimethylation, alleviates Polycomb‐mediated repression and sensitizes AML cells to retinoic acid.^51^ With downregulation of E2F1 and CDK1 enforcing G1 arrest, this allows differentiation programmes to proceed.^52^

To identify potential predictors of therapeutic response in primary AML samples, we sought to correlate the mutation status of known oncogenes, such as FLT3‐ITD, NPM1 and CEBPA, or FAB classification with treatment response. This analysis, likely due to its limited sample size, did not reveal any significant correlations.

We therefore examined pretreatment gene expression in primary AML samples. We identified specific genes correlating with treatment response, including LTF, CRISP3, OLFM4, CEACAM5 and PRUNE2, which are crucial for neutrophil functions. LTF, found in neutrophil secondary granules, is a potential prognostic indicator in AML.^53^ CRISP3 regulates neutrophil migration and antimicrobial response.^54^ OLFIM4 overexpression in HL‐60 cells leads to growth inhibition, differentiation, apoptosis and enhanced ATRA response.^55^ PRUNE2^56^ is a promising candidate gene for predicting AML treatment response, involved in tumour pathogenesis and shown to inhibit cell proliferation while promoting apoptosis.^57^ Pretreatment expression of these genes may predict response to ATRA and palbociclib treatment, though further research is needed.

In summary, our findings indicate that the combination of palbociclib and ATRA facilitates differentiation and may represent a promising strategy in AML treatment. Overall, dual targeting of the cell cycle regulators in combination with oncogenic signalling pathways represents an effective approach to overcome the differentiation blockade in AML. Given that palbociclib is already FDA‐approved and in clinical use, our findings hold significant clinical relevance, suggesting its potential use for AML management. Further translational and clinical research would be warranted to elucidate the complex mechanism of efficacy and to identify patients who could potentially benefit from treatment.

## AUTHOR CONTRIBUTIONS

*Conceptualization and supervision*: T.S., L.S. and A.Z. *Methodology*: S.M., J.S., F.G.Z., S.G.Ö., P.C., M.P., S.M. and R.S. *Investigation*: S.M., R.S., F.G.Z., C.S., K.M.G., S.G.Ö. and A.B. *Data curation*: J.S., C.S., S.M., A.B. and P.C. *Funding acquisition*: T.S., L.S., M.F. and A.Z. *Writing—original draft preparation*: R.S., L.S., S.G.Ö., P.C. and T.S. *Writing—review and editing*: M.F., K.M.G., Y.S., S.S., L.S., A.B. and P.C. *Visualization*: S.M., R.S. and S.S. All authors have read and agreed to the published version of the manuscript.

## FUNDING INFORMATION

R.S., M.P., A.Z. and L.S. were supported by Polish National Science Centre grant no. 2019/33/B/NZ5/02399 and K.M.G. was supported by Polish National Science Centre grant no. 2021/41/B/NZ5/04397. F.G.Z. was supported by the Landesgraduiertenstipendium of the State of Thuringia. C.S. and T.S. were supported by the German Research Council (SCHE1909/2‐3). The work of S.G.Ö., M.F. and T.S. was supported by the Carl‐Zeiss‐Stiftung (Carl Zeiss Foundation) through the Jena School of Molecular Medicine.

## CONFLICT OF INTEREST STATEMENT

The authors declare no conflict of interest.

## Supporting information


Data S1.

## Data Availability

The data that support the findings of this study are available from the corresponding authors, upon reasonable request. High‐throughput data discussed in this publication were deposited in NCBI's Gene Expression Omnibus and are accessible through GEO Series accession numbers GSE291868 and GSE260773.

## References

[1] Hou H‐A , Tien H‐F . Genomic landscape in acute myeloid leukemia and its implications in risk classification and targeted therapies. J Biomed Sci. 2020;27(1):81. 32690020 10.1186/s12929-020-00674-7 PMC7372828

[2] Hwang SM . Classification of acute myeloid leukemia. Blood Res. 2020;55(S1):S1–S4. 32719169 10.5045/br.2020.S001 PMC7386892

[3] Park HS . What is new in acute myeloid leukemia classification? Blood Res. 2024;59(1):15. 38616211 10.1007/s44313-024-00016-8 PMC11016528

[4] Choi JK , Xiao W , Chen X , Loghavi S , Elenitoba‐Johnson KS , Naresh KN , et al. Of the World Health Organization classification of tumors of the hematopoietic and lymphoid tissues. Mod Pathol. 2024;37:100466. 38460674 10.1016/j.modpat.2024.100466

[5] DiNardo CD , Cortes JE . Mutations in AML: prognostic and therapeutic implications. Hematology Am Soc Hematol Educ Program. 2016;2016:348–355. 27913501 10.1182/asheducation-2016.1.348 PMC6142505

[6] Bazarbachi A , Schmid C , Labopin M , Beelen D , Wolfgang Blau I , Potter V , et al. Evaluation of trends and prognosis over time in patients with AML relapsing after allogeneic hematopoietic cell transplant reveals improved survival for young patients in recent years. Clin Cancer Res. 2020;26(24):6475–6482. 32988970 10.1158/1078-0432.CCR-20-3134

[7] Oliva EN , Franek J , Patel D , Zaidi O , Nehme SA , Almeida AM . The real‐world incidence of relapse in acute myeloid leukemia (AML): A systematic literature review (SLR). Blood. 2018;132(Supplement 1):5188.

[8] Patel A , Agha M , Raptis A , Hou JZ , Farah R , Redner RL , et al. Outcomes of patients with acute myeloid leukemia who relapse after 5 years of complete remission. Oncol Res. 2021;28(7):811–814. 32753091 10.3727/096504020X15965357399750 PMC8420904

[9] Reville PK , Nogueras González GM , Ravandi F , Sasaki K , Borthakur G , Garcia‐Manero G , et al. Predictors of early mortality, response, and survival in newly diagnosed acute myeloid leukemia (AML) using a contemporary academic cohort. Blood. 2020;136:44–45.

[10] Lo‐Coco F , Avvisati G , Vignetti M , Thiede C , Orlando SM , Iacobelli S , et al. Retinoic acid and arsenic trioxide for acute promyelocytic leukemia. N Engl J Med. 2013;369(2):111–121. 23841729 10.1056/NEJMoa1300874

[11] Sanz MA , Fenaux P , Tallman MS , Estey EH , Löwenberg B , Naoe T , et al. Management of acute promyelocytic leukemia: updated recommendations from an expert panel of the European LeukemiaNet. Blood. 2019;133(15):1630–1643. 30803991 10.1182/blood-2019-01-894980 PMC6509567

[12] Lampen A , Meyer S , Arnhold T , Nau H . Metabolism of vitamin A and its active metabolite all‐trans‐retinoic acid in small intestinal enterocytes. J Pharmacol Exp Ther. 2000;295(3):979–985. 11082432

[13] Cañete A , Cano E , Muñoz‐Chápuli R , Carmona R . Role of vitamin A/retinoic acid in regulation of embryonic and adult hematopoiesis. Nutrients. 2017;9(2):159. 28230720 10.3390/nu9020159 PMC5331590

[14] McKenna NJ . EMBO Retinoids 2011: mechanisms, biology and pathology of signaling by retinoic acid and retinoic acid receptors. Nucl Recept Signal. 2012;10:e003. 22438793 10.1621/nrs.10003 PMC3309077

[15] Schenk T , Stengel S , Zelent A . Unlocking the potential of retinoic acid in anticancer therapy. Br J Cancer. 2014;111(11):2039–2045. 25412233 10.1038/bjc.2014.412 PMC4260020

[16] Castelijn DAR , Sijm G , Venniker‐Punt B , Poddighe PJ , Wondergem MJ . An acute promyelocytic leukemia resistant to all‐trans retinoic acid: a case report of the ZBTB16::RARa variant and review of the literature. Case Rep Oncol. 2023;16(1):1443–1450. 38028572 10.1159/000534862 PMC10666957

[17] Schenk T , Chen WC , Göllner S , Howell L , Jin L , Hebestreit K , et al. Inhibition of the LSD1 (KDM1A) demethylase reactivates the all‐trans‐retinoic acid differentiation pathway in acute myeloid leukemia. Nat Med. 2012;18(4):605–611. 22406747 10.1038/nm.2661 PMC3539284

[18] Stengel S , Petrie KR , Sbirkov Y , Stanko C , Ghazvini Zadegan F , Gil V , et al. Suppression of MYC by PI3K/AKT/mTOR pathway inhibition in combination with all‐trans retinoic acid treatment for therapeutic gain in acute myeloid leukaemia. Br J Haematol. 2022;198(2):338–348. 35468223 10.1111/bjh.18187

[19] Su M , Alonso S , Jones JW , Yu J , Kane MA , Jones RJ , et al. All‐trans retinoic acid activity in acute myeloid leukemia: role of cytochrome P450 enzyme expression by the microenvironment. PLoS One. 2015;10(6):e0127790. 26047326 10.1371/journal.pone.0127790 PMC4457893

[20] Alvares CL , Schenk T , Hulkki S , Min T , Vijayaraghavan G , Yeung J , et al. Tyrosine kinase inhibitor insensitivity of non‐cycling CD34+ human acute myeloid leukaemia cells with FMS‐like tyrosine kinase 3 mutations. Br J Haematol. 2011;154(4):457–465. 21689085 10.1111/j.1365-2141.2011.08748.x

[21] Kahl M , Brioli A , Bens M , Perner F , Kresinsky A , Schnetzke U , et al. The acetyltransferase GCN5 maintains ATRA‐resistance in non‐APL AML. Leukemia. 2019;33(11):2628–2639. 31576004 10.1038/s41375-019-0581-y

[22] Eisfeld AK , Kohlschmidt J , Schwind S , Nicolet D , Blachly JS , Orwick S , et al. Mutations in the CCND1 and CCND2 genes are frequent events in adult patients with t(8;21)(q22;q22) acute myeloid leukemia. Leukemia. 2017;31(6):1278–1285. 27843138 10.1038/leu.2016.332 PMC5462855

[23] Faber ZJ , Chen X , Gedman AL , Boggs K , Cheng J , Ma J , et al. The genomic landscape of core‐binding factor acute myeloid leukemias. Nat Genet. 2016;48(12):1551–1556. 27798625 10.1038/ng.3709 PMC5508996

[24] Baker SJ , Poulikakos PI , Irie HY , Parekh S , Reddy EP . CDK4: a master regulator of the cell cycle and its role in cancer. Genes Cancer. 2022;13:21–45. 36051751 10.18632/genesandcancer.221 PMC9426627

[25] Morrison L , Loibl S , Turner NC . The CDK4/6 inhibitor revolution—a game‐changing era for breast cancer treatment. Nat Rev Clin Oncol. 2024;21(2):89–105. 38082107 10.1038/s41571-023-00840-4

[26] Hortobagyi GN , Stemmer SM , Burris HA , Yap Y‐S , Sonke GS , Hart L , et al. Overall survival with ribociclib plus letrozole in advanced breast cancer. N Engl J Med. 2022;386(10):942–950. 35263519 10.1056/NEJMoa2114663

[27] Cicenas J , Kalyan K , Sorokinas A , Jatulyte A , Valiunas D , Kaupinis A , et al. Highlights of the latest advances in research on CDK inhibitors. Cancers (Basel). 2014;6(4):2224–2242. 25349887 10.3390/cancers6042224 PMC4276963

[28] Ryu C‐K , Kang H‐Y , Lee SK , Nam KA , Hong CY , Ko W‐G , et al. 5‐Arylamino‐2‐methyl‐4,7‐dioxobenzothiazoles as inhibitors of cyclin‐dependent kinase 4 and cytotoxic agents. Bioorg Med Chem Lett. 2000;10(5):461–464. 10743948 10.1016/s0960-894x(00)00014-7

[29] Drusbosky LM , Turcotte M , Castillo P , Vidva R , Gera S , Sauban M , et al. Predicting response to CDK4/6 inhibitors and combinations using a computational biology model and its validation: a beat AML project study. Blood. 2017;130(Supplement 1):3909.

[30] Nakatani K , Matsuo H , Harata Y , Higashitani M , Koyama A , Noura M , et al. Inhibition of CDK4/6 and autophagy synergistically induces apoptosis in t(8;21) acute myeloid leukemia cells. Int J Hematol. 2021;113(2):243–253. 33068248 10.1007/s12185-020-03015-4

[31] Xie X , Zhang W , Zhou X , Ye Z , Wang H , Qiu Y , et al. Abemaciclib drives the therapeutic differentiation of acute myeloid leukaemia stem cells. Br J Haematol. 2023;201(5):940–953. 36916190 10.1111/bjh.18735

[32] Hanahan D . Hallmarks of cancer: new dimensions. Cancer Discov. 2022;12(1):31–46. 35022204 10.1158/2159-8290.CD-21-1059

[33] Topacio BR , Zatulovskiy E , Cristea S , Xie S , Tambo CS , Rubin SM , et al. Cyclin D‐Cdk4,6 drives cell‐cycle progression via the retinoblastoma protein's C‐terminal helix. Mol Cell. 2019;74(4):758–770.e4. 30982746 10.1016/j.molcel.2019.03.020 PMC6800134

[34] Burnett AK , Hills RK , Green C , Jenkinson S , Koo K , Patel Y , et al. The impact on outcome of the addition of all‐trans retinoic acid to intensive chemotherapy in younger patients with nonacute promyelocytic acute myeloid leukemia: overall results and results in genotypic subgroups defined by mutations in NPM1, FLT3, and CEBPA. Blood. 2010;115(5):948–956. 19965647 10.1182/blood-2009-08-236588

[35] Küley‐Bagheri Y , Kreuzer KA , Monsef I , Lübbert M , Skoetz N . Effects of all‐trans retinoic acid (ATRA) in addition to chemotherapy for adults with acute myeloid leukaemia (AML) (non‐acute promyelocytic leukaemia (non‐APL)). Cochrane Database Syst Rev. 2018;8(8):Cd011960. 30080246 10.1002/14651858.CD011960.pub2 PMC6513628

[36] Schlenk RF , Lübbert M , Benner A , Lamparter A , Krauter J , Herr W , et al. All‐trans retinoic acid as adjunct to intensive treatment in younger adult patients with acute myeloid leukemia: results of the randomized AMLSG 07‐04 study. Ann Hematol. 2016;95(12):1931–1942. 27696203 10.1007/s00277-016-2810-z PMC5093206

[37] Uras IZ , Walter GJ , Scheicher R , Bellutti F , Prchal‐Murphy M , Tigan AS , et al. Palbociclib treatment of FLT3‐ITD+ AML cells uncovers a kinase‐dependent transcriptional regulation of FLT3 and PIM1 by CDK6. Blood. 2016;127(23):2890–2902. 27099147 10.1182/blood-2015-11-683581 PMC4920675

[38] Placke T , Faber K , Nonami A , Putwain SL , Salih HR , Heidel FH , et al. Requirement for CDK6 in MLL‐rearranged acute myeloid leukemia. Blood. 2014;124(1):13–23. 24764564 10.1182/blood-2014-02-558114 PMC4190617

[39] Graña X , Reddy EP . Cell cycle control in mammalian cells: role of cyclins, cyclin dependent kinases (CDKs), growth suppressor genes and cyclin‐dependent kinase inhibitors (CKIs). Oncogene. 1995;11(2):211–219. 7624138

[40] Kato JY , Matsuoka M , Strom DK , Sherr CJ . Regulation of cyclin D‐dependent kinase 4 (cdk4) by cdk4‐activating kinase. Mol Cell Biol. 1994;14(4):2713–2721. 8139570 10.1128/mcb.14.4.2713-2721.1994 PMC358637

[41] Fischer M , Schade AE , Branigan TB , Müller GA , DeCaprio JA . Coordinating gene expression during the cell cycle. Trends Biochem Sci. 2022;47(12):1009–1022. 35835684 10.1016/j.tibs.2022.06.007

[42] Schnerch D , Yalcintepe J , Schmidts A , Becker H , Follo M , Engelhardt M , et al. Cell cycle control in acute myeloid leukemia. Am J Cancer Res. 2012;2(5):508–528. 22957304 PMC3433102

[43] Aref S , Mabed M , El‐Sherbiny M , Selim T , Metwaly A . Cyclin D1 expression in acute leukemia. Hematology. 2006;11(1):31–34. 16522546 10.1080/10245330500322321

[44] Jaroslav P , Martina H , Jirí S , Hana K , Petr S , Tomás K , et al. Expression of cyclins D1, D2, and D3 and Ki‐67 in leukemia. Leuk Lymphoma. 2005;46(11):1605–1612. 16334487 10.1080/10428190500215100

[45] Scheicher R , Hoelbl‐Kovacic A , Bellutti F , Tigan AS , Prchal‐Murphy M , Heller G , et al. CDK6 as a key regulator of hematopoietic and leukemic stem cell activation. Blood. 2015;125(1):90–101. 25342715 10.1182/blood-2014-06-584417 PMC4281832

[46] Drach J , Lopez‐Berestein G , McQueen T , Andreeff M , Mehta K . Induction of differentiation in myeloid leukemia cell lines and acute promyelocytic leukemia cells by liposomal all‐trans‐retinoic acid. Cancer Res. 1993;53(9):2100–2104. 8481912

[47] Hu L , Li Q , Wang J , Wang H , Ren X , Huang K , et al. The CDK4/6 inhibitor palbociclib synergizes with ATRA to induce differentiation in AML. Mol Cancer Ther. 2024;23(7):961–972. 38507743 10.1158/1535-7163.MCT-23-0528

[48] Qu W , Lu J , Ji Y , He Z , Hou M , Li D , et al. Successful use of palbociclib combined with venetoclax and azacitidine in an adult with refractory/relapsed therapy‐related acute myeloid leukemia. Cancer Chemother Pharmacol. 2024;94(4):635–639. 38430306 10.1007/s00280-024-04642-y

[49] Lagunas‐Rangel FA . KDM6B (JMJD3) and its dual role in cancer. Biochimie. 2021;184:63–71. 33581195 10.1016/j.biochi.2021.02.005

[50] Bannister AJ , Falcão AM , Castelo‐Branco G . Histone modifications and histone variants in pluripotency and differentiation. In: Göndör A , editor. Chromatin regulation and dynamics. Cambridge, MA: Elsevier; 2017. p. 35–64.

[51] Sbirkov Y , Schenk T , Kwok C , Stengel S , Brown R , Brown G , et al. Dual inhibition of EZH2 and G9A/GLP histone methyltransferases by HKMTI‐1‐005 promotes differentiation of acute myeloid leukemia cells. Front Cell Dev Biol. 2023;11:1076458. 37035245 10.3389/fcell.2023.1076458 PMC10076884

[52] Radomska HS , Alberich‐Jorda M , Will B , Gonzalez D , Delwel R , Tenen DG . Targeting CDK1 promotes FLT3‐activated acute myeloid leukemia differentiation through C/EBPalpha. J Clin Invest. 2012;122(8):2955–2966. 22797303 10.1172/JCI43354 PMC3408728

[53] Chen T , Zhang J , Wang Y , Zhou H . Identification of survival‐related genes in acute myeloid leukemia (AML) based on cytogenetically normal AML samples using weighted gene coexpression network analysis. Dis Markers. 2022;2022(1):5423694. 36212177 10.1155/2022/5423694 PMC9537620

[54] Jurgec S , Jezernik G , Gorenjak M , Büdefeld T , Potočnik U . Meta‐analytic comparison of global RNA transcriptomes of acute and chronic myeloid leukemia cells reveals novel gene candidates governing myeloid malignancies. Cancer. 2022;14(19):4681. 10.3390/cancers14194681 PMC9562668 36230605

[55] Liu W , Lee HW , Liu Y , Wang R , Rodgers GP . Olfactomedin 4 is a novel target gene of retinoic acids and 5‐aza‐2'‐deoxycytidine involved in human myeloid leukemia cell growth, differentiation, and apoptosis. Blood. 2010;116(23):4938–4947. 20724538 10.1182/blood-2009-10-246439 PMC3012588

[56] Zhu R , Lin W , Tang L , Hu Y . Identification of hub genes associated with adult acute myeloid leukemia progression through weighted gene co‐expression network analysis. Aging (Albany NY). 2021;13(4):5686–5697. 33592582 10.18632/aging.202493 PMC7950274

[57] Li T , Huang S , Yan W , Zhang Y , Guo Q . FOXF2 regulates PRUNE2 transcription in the pathogenesis of colorectal cancer. Technol Cancer Res Treat. 2022;21:15330338221118717. 35929169 10.1177/15330338221118717 PMC9358570

