# Supplementary material for: Synergistic potential of CDK4/6 inhibitors and ATRA in non‐APL AML
Source: Br J Haematol. 2025 Aug 4;207(4):1279–88. doi: 10.1111/bjh.70057 (PMC12512083; doi:10.1111/bjh.70057)
Supplement: Supplementary file 1 — Data S1. [file BJH-207-1279-s001.docx]

**Synergistic Potential of CDK4/6 Inhibitors and ATRA in non-APL AML.**

**Supplementary Materials and Methods**

**Supplementary Figures**

- Supplementary Figure 1: *Differentiation and cell cycle analysis (Ryuvidine)*
- Supplementary Figure 2: *Differentiation and cell cycle analysis (CDK4i)*
- Supplementary Figure 3: *Correlation mRNA levels/response in primary AML*
- Supplementary Figure 4: *Cell viability (Ryuvidine)*

**Supplementary Tables**

- Supplementary Table 1: *CDI calculations (Palbociclib + ATRA)*
- Supplementary Table 2: *CDI calculations (Ryuvidine + ATRA)*
- Supplementary Table 3: *Patient Characteristics*

## Materials and methods

Antibodies used for immunoblotting

For immunoblotting, following primary antibodies were used: Anti-phospho-Rb (Ser807/811) (#8516, Cell Signaling Technology; 1:1000), anti-Rb (#9309, Cell Signaling Technology; 1:1000), anti-β-actin (A5316, Sigma-Aldrich; 1:5000). For the detection, 1:10000 secondary antibodies goat anti-rabbit (#5220-0336, SeraCare) and goat anti-mouse (#5220-0341, SeraCare) were used.

RNAseq analysis

HL-60 cells were single or double-treated (3 hours; 500 nM palbociclib, 100 nM ATRA) and total RNA was isolated. TruSeq RNA Library preparation was performed using oligo(dt) beads binding all mRNAs by their polyadenylic (polyA) tail. Barcoded triplicates of each treatment condition including untreated were sequenced using Illumina NGS (HiSeq 2500). Sequencing data was uploaded in fastq format into Galaxy (32) and read quality was checked using the FASTQC tool (av. read no per sample: 21 +/- 3mio, av. percentage of basecalls Q30-40: >94%). Reads were mapped against the human genome (hg38) using HISAT2 and counts were quantified via the htseq-count program with default settings (33, 34). The mapped count files generated were uploaded to Degust ([https://degust.erc.monash.edu](https://degust.erc.monash.edu/)) to perform differential gene analysis using the Voom method (PMID: 24485249). Multidimensional scaling (MDS) and was employed to characterize the effects of ATRA and palbociclib. Hierarchical clustering analysis was performed using Morpheus (clue.io/morpheus).

For the RNA sequencing of patient samples, peripheral blood samples were obtained from 28 AML patients prior to treatment. Following PBMC isolation, total RNA isolation, library preparation and NGS were performed as described. Principal component analysis (PCA) was conducted on the mRNA sequencing libraries to examine gene similarities and differences associated with interpersonal variability. Pearson’s correlation coefficient was used to assess the relationship between baseline gene expression and differentiation, as indicated by the expression levels of CD11b.

*Statistical analysis*

Data are represented as the mean ± standard deviation (SD) and were generated from three independent experiments unless otherwise specified. Statistical analysis was performed using two-way ANOVA with Bonferroni post-test using GraphPad Prism 10 software. Statistical significance was defined as P<0.05 (*P<0.05; **P<0.01; ***P<0.001). RNA sequencing data from patient samples were sorted by expression, and only genes that were expressed in at least two samples were included in the analysis. Pearson’s correlation coefficient was used to determine the correlation between baseline expression and differentiation. Expression correlations between individual genes that might affect the analysis are presented as a heat map. Given the observed correlations in gene expression, an additional correlation analysis was conducted to ensure the integrity of the multivariate analysis results. Correlation analysis identified four clusters of genes with correlated expression patterns. Consequently, a multivariate analysis was performed with these four clusters in consideration, rather than focusing on individual gene expression. Multivariate analysis was performed using a Cox proportional hazards regression model. Statistical analysis was performed using R version 4.3.2 (2).

## Supplementary Figures


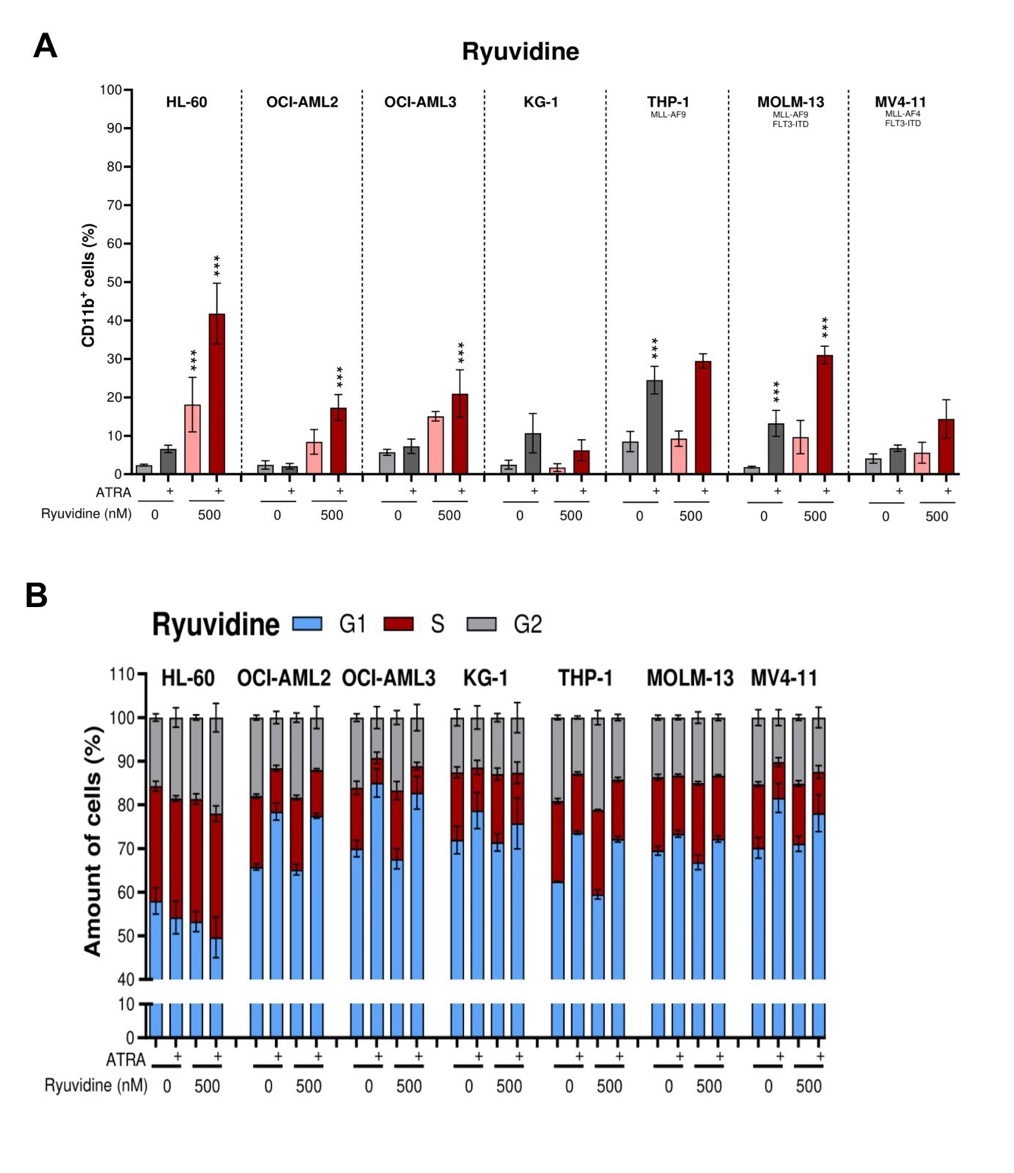


**Supplementary Figure 1.**

(A) Flow cytometric analysis of differentiation marker CD11b expression in AML cell lines treated with 500 nM ryuvidine, ATRA (10 nM for THP-1, 1000 nM for KG-1, and 100 nM for the remaining cell lines) or incombination. (B) Flow cytometric analysis of cell cycle in AML cell lines treated with 500 nM ryuvidine, ATRA (10 nM for THP-1, 1000 nM for KG-1, and 100 nM for the remaining cell lines) or in combination. Statistical analyses were performed by comparing combined treatments to single ATRA treatment, whereas single palbociclib and single ATRA treatments were compared to untreated samples. (mean ± SD, n=3, Two-way ANOVA, Bonferroni correction)


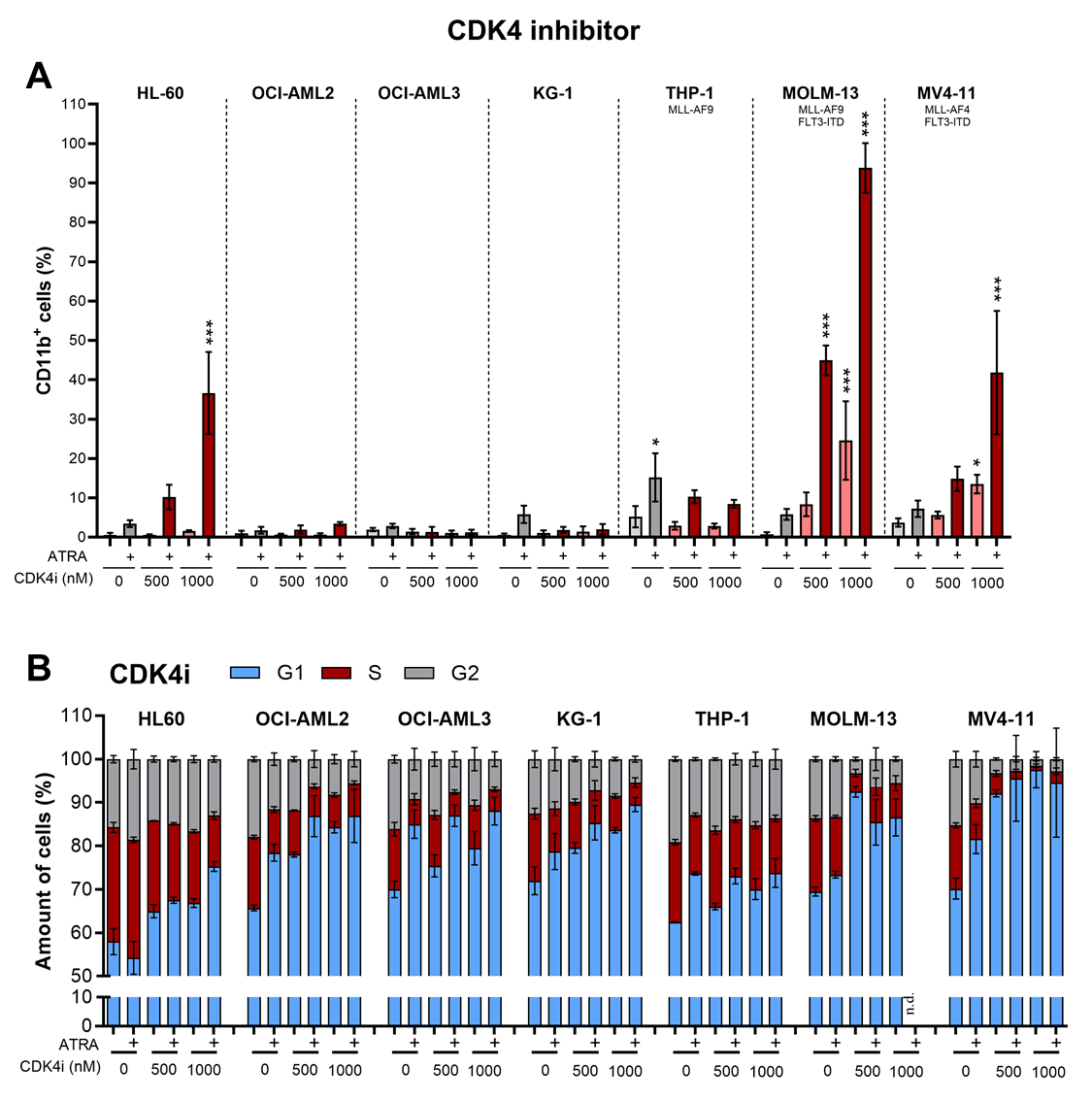


**Supplementary Figure 2.**

(A) Flow cytometric analysis of differentiation marker CD11b expression in AML cell lines treated with 500 or 1000 nM CDK4 inhibitor, ATRA (10 nM for THP-1, 1000 nM for KG-1, and 100 nM for the remaining cell lines) or incombination. (B) Flow cytometric analysis of cell cycle in AML cell lines treated with 500 or 1000 nM CDK4 inhibitor, ATRA (10 nM for THP-1, 1000 nM for KG-1, and 100 nM for the remaining cell lines) or in combination. Statistical analyses were performed by comparing combined treatments to single ATRA treatment, whereas single palbociclib and single ATRA treatments were compared to untreated samples. (mean ± SD, n=3, Two-way ANOVA, Bonferroni correction)


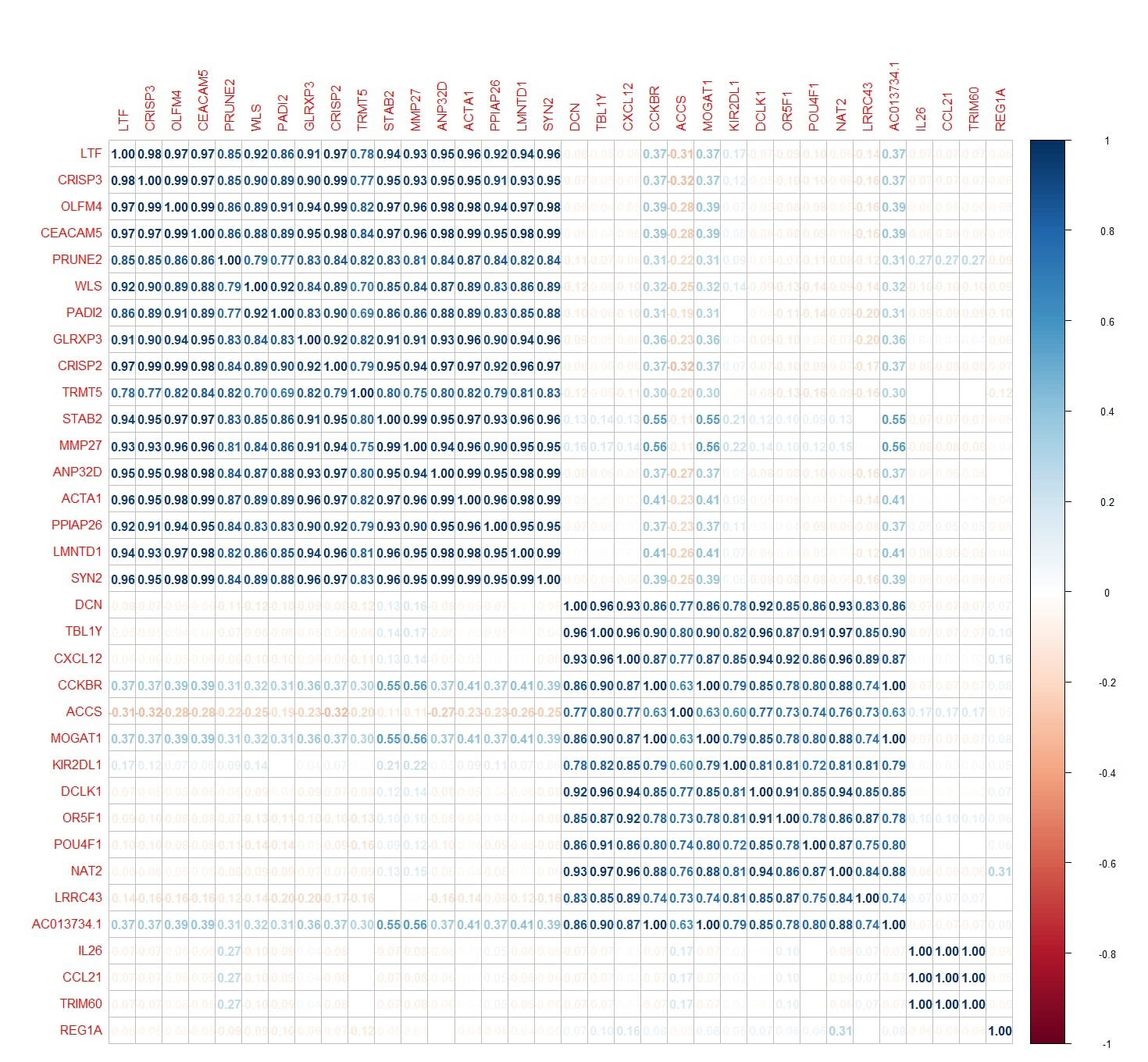


**Supplementary Figure 3.**

Heatmap presenting the results of a gene expression analysis conducted on patient samples. A total of 34 genes were expressed in at least two samples and were thus included in the subsequent analysis. The analyzed pretreatment genes exhibited a high level of correlation, thereby enabling the identification of four groups of the most correlated genes through the use of the heatmap. A high level of positive correlation is indicated by positive numbers (blue). The defined groups included the following genes: group 1: *LTF, CRISP3, OLFM4, CEACAM5, PRUNE2, MLS, PADI2, GLRXP3, CRISP2, TRMT5, STAB2, MMP27, AMP32D, ACTA1, PPIAP26, LMNTD1*, and *SYN2*; group 2: *DCN, TBL1Y, CXCL12, CCKBR, ACCS, MOGAT1, KIR2DL1, DCLK1, OR5F1, POU4F1, NAT2, LRRC43*, and *AC013734.1*, group 3: *IL26, CCL21*, and *TRIM*. Group 4 containing one *REG1A* gene uncorrelated in expression with others. A correlation between Group 1 of genes and differentiation defined by increased CD11b expression was found (p < 0.005).

**Supplementary Figure 4.**
Cell viability analysis of AML cell lines treated with ryuvidine and ATRA. AML cell lines were treated with ryuvidine and ATRA for 3 days and MTT assay was performed. Absorbance was measured at 570 nm. IC50 values of single ryuvidine treatment and combination treatment are indicated. (mean ± SD, n = 3; each experiment performed as triplicates, normalized to untreated)

**Supplementary Table 1**: CDI calculations for Palbociclib + ATRA treatment of AML cell lines.

***
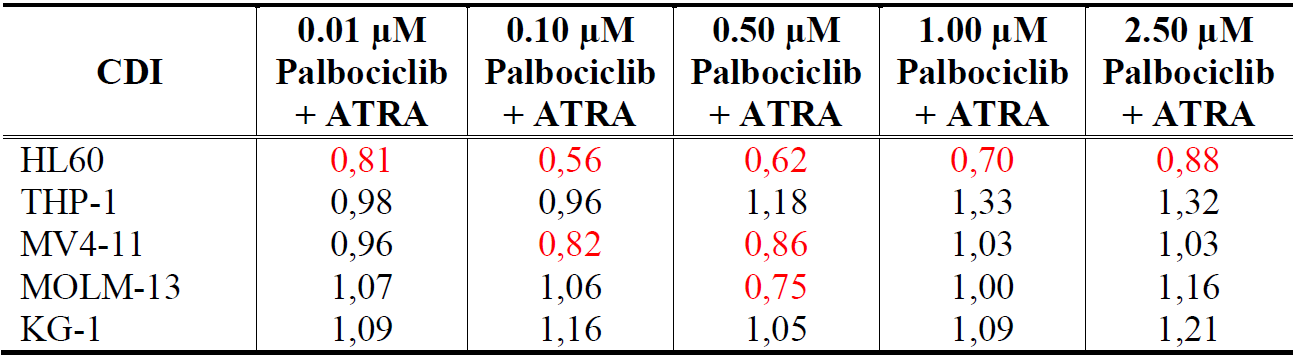
***

CDI < 1 synergistic effect, CDI = 1 additive effect, CDI > 1 antagonistic effect. CDI values considered

as synergistic were highlighted in red.

**Supplementary Table 2**: CDI calculations for Ryuvidine + ATRA treatment of AML cell lines.

***
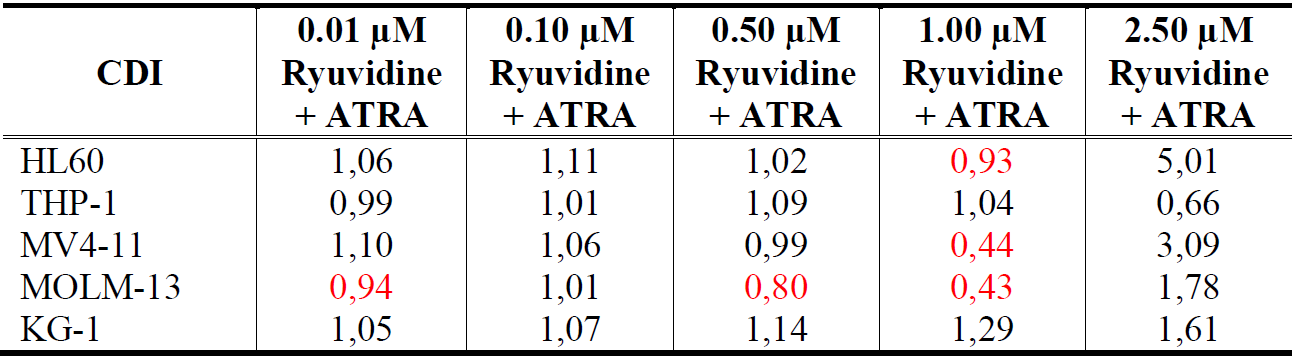
***

CDI < 1 synergistic effect, CDI = 1 additive effect, CDI > 1 antagonistic effect. CDI values considered

as synergistic were highlighted in red.

**Supplementary Table 3: Patient Characteristics**

| **Characteristics** | **Patients (N=39)** |
| --- | --- |
| **Median age (range)** | 64 years (37 to 86 years) |
| **Sex (m/f)** | 16/23 |
| **FAB (%)** |  |
| M0 | 1 (3) |
| M1 | 3 (8) |
| M2 | 6 (15) |
| M4 | 3 (8) |
| M5 | 4 (10) |
| M6 | 1 (3) |
| Not classified | 21 (54) |
| **Type (%)** |  |
| Primary | 13 (33) |
| Secondary | 6 (15) |
| Relapsed | 6 (15) |
| Not classified | 14 (36) |
| **Blasts in peripheral blood** |  |
| >50% | 13 (33) |
| <50% | 11 (28) |
| Not determined | 15 (38) |
| **Cytogenetics (%)** |  |
| normal | 10 (26) |
| complex | 2 (5) |
| t8;21 | 4 (10) |
| Not tested  Other | 17 (44)  6 (15) |
| **Mutations (% tested positive)** |  |
| FLT3-ITD | 10 (26) |
| NPM1 | 6 (15) |
| RUNX1-RUNX1T1 | 4 (10) |
| CEBPA | 1 (3) |
| KMT2A-PTD | 2 (5) |
| PML-RARA | 0 (0) |
| Other  Not tested / Unclassified | 10 (26)  18 (46) |

**References:**

1. Schenk T, Chen WC, Göllner S, Howell L, Jin L, Hebestreit K, et al. Inhibition of the LSD1 (KDM1A) demethylase reactivates the all-trans-retinoic acid differentiation pathway in acute myeloid leukemia. Nat Med. 2012;18(4):605-11.

2. Therneau TM. A Package for Survival Analysis in R.
